# Smart Supramolecular Self-Assembled Nanosystem: Stimulus-Responsive Hydrogen-Bonded Liquid Crystals

**DOI:** 10.3390/nano11020448

**Published:** 2021-02-10

**Authors:** Bing Liu, Tao Yang, Xin Mu, Zhijian Mai, Hao Li, Yao Wang, Guofu Zhou

**Affiliations:** 1 Guangdong Provincial Key Laboratory of Optical Information Materials and Technology & Institute of Electronic Paper Displays, South China Academy of Advanced Optoelectronics, South China Normal University, Guangzhou 510006, China; liubingscnu2017@163.com (B.L.); yangtao19960303@163.com (T.Y.); muxin_y@163.com (X.M.); maizhijian0504@163.com (Z.M.); guofu.zhou@m.scnu.edu.cn (G.Z.); 2 National Center for International Research on Green Optoelectronics, South China Normal University, Guangzhou 510006, China

**Keywords:** liquid crystal, hydrogen bonds, supramolecular, self-assembled, stimulus-responsive

## Abstract

In a liquid crystal (LC) state, specific orientations and alignments of LC molecules produce outstanding anisotropy in structure and properties, followed by diverse optoelectronic functions. Besides organic LC molecules, other nonclassical components, including inorganic nanomaterials, are capable of self-assembling into oriented supramolecular LC mesophases by non-covalent interactions. Particularly, huge differences in size, shape, structure and properties within these components gives LC supramolecules higher anisotropy and feasibility. Therefore, hydrogen bonds have been viewed as the best and the most common option for supramolecular LCs, owing to their high selectivity and directionality. In this review, we summarize the newest advances in self-assembled structure, stimulus-responsive capability and application of supramolecular hydrogen-bonded LC nanosystems, to provide novel and immense potential for advancing LC technology.

## 1. Introduction

As a well-known material, liquid crystal (LC) unites molecular ordering and mobility [1,2,3,4,5,6]. The LC state of matter exists between the solid and isotropic liquid phase, and is therefore defined as a mesophase [7,8,9]. Sometimes it is referred to “the fourth state of matter”. In 1888, Friedrich Reinitzer found a cholesterol ester which had “two melting points”, the cholesterol ester firstly melted into a cloudy liquid at 145.5 °C, and became transparent at 178.5 °C [10]. Furthermore, O. Lehmann named this kind of material “liquid crystal”, with both liquid fluidity and crystal optical anisotropy [11].

According to the forming condition of the mesophase, LC can usually be divided into thermotropic and lyotropic LC [12]. Typically, thermotropic LC mesophase appears within a certain temperature range without any additive, but lyotropic mesophase needs to gain enough fluidity or mobility through interactions with solvent molecules, e.g., soaps. Given different mesophases, thermotropic LC is further classified into nematic, cholesteric and smectic phases. This classification, created by Georges Friedel the 19th century, depends on the molecular arrangement [13]. 

During the last few decades, there has been a lot of progress in interdisciplinary developments of LC materials and applications, e.g., organic electronics, templates, reflectors, actuators, sensors and nanoporous films [3,14,15]. In order to advance performance, multifunctional supramolecular materials have gradually developed into a novel platform for LC self-assemblies with a large scale and high anisotropy [16].

## 2. Nature of Liquid Crystal Molecules: Anisotropy

In nature, there are abundant materials with physicochemical anisotropy. When part or all of the physicochemical properties of a substance varies with directional change, this substance is defined as anisotropic. LC molecules are considered as the most typical anisotropic materials.

The anisotropy of physical properties, including refractive index, dielectric constant, viscosity and so on, make LC materials special. Unlike isotropic phase, the LC phase shows a macroscopic anisotropy featuring non-zero average value of these properties in different directions [17]. Microscopically, this is a consequence of specific electronic and steric interactions within LC molecules. These interactions lead to long-range intermolecular order, which makes LCs significantly different from ordinary isotropic liquids [18].

On account of this unique anisotropic performance, LC materials are very sensitive to external physical stimulus, including light, electricity, magnetism and so on. Typically, LC optical and dielectric anisotropy play a key role in display technology. Nowadays, stimulus-responsive LC materials have been made, with big developments in many fields, e.g., actuators, sensors, ion transport and templates.

For example, one of the main LC characteristics in optics is birefringence, namely, anisotropy in the refractive index, which behaves similarly to optical uniaxial crystal. As LC material develops under suitable conditions (e.g., temperature or solvent), some optical textures of birefringence can be observed by a polarizing optical microscope, like striped texture, planar texture, focal conic texture, fingerprint-like texture and mosaic texture [19]. This is the simplest way to determine whether a substance has an LC state. 

Taking a rod-shaped LC molecule as an example, it can be seen that it is a uniaxial crystal. The uniaxial crystal has two different refractive indexes, *n_o_* and *n_e_*. *n_o_* is ordinary light, and its electric vector vibration direction is perpendicular to the optical axis of the LC molecule; *n_e_* represents extraordinary light, whose electric vector vibration direction is parallel to the optical axis of the LC molecule. So, the optical anisotropy (Δ*n*) is expressed as:

Δ*n* = *n*_e_ − *n*_o_.

Another important characteristic of LC is dielectric anisotropy (Δε). For LC display devices, the threshold voltage is a key parameter and mainly depends on dielectric anisotropy. The optical anisotropy (Δε) is expressed as:

Δε = ε_*∥*_ − ε*_*⊥*_*.

where ε_*∥*_ and ε*_⊥_* denote the dielectric constant measured in the applied electric field parallel and perpendicular to the optical axis of the liquid crystal, respectively.

In a word, special anisotropy makes LC attract a lot of attention since different LC materials show macroscopic changes in different directions or when exposed to an external stimulus [20]. 

## 3. Supramolecular Liquid Crystals 

When alkali metal cations and several types of strong and highly selective ligands were discovered in 1960s, supramolecular chemistry began to grow into an independent discipline [21,22,23]. In 1987, Lehn defined supramolecular chemistry as the science which focuses on the structure and function of supramolecular systems formed by intermolecular forces of two or more compounds [24].

In 1927, E. Bradfield and B. Jones found the first supramolecular LC structure generated by hydrogen bonding between the carboxyl groups of benzoic acid derivatives [1,21]. Obviously, supramolecular LC is totally different from traditional covalent-bonded LC molecules, and should be viewed as an LC composite system based on intermolecular non-covalent interactions, including hydrogen bonds (H-bonds), halogen bonds, van der Waals force, electrostatic interaction, conjugation effect, hydrophobic interaction and so on. To date, supramolecular LC has gradually developed into a mature and widely applied discipline for lots of aspects (e.g., nanowire [25,26,27,28], templates [29], LC physical gel [30] and electro-optic materials [31,32]).

Compared with covalent bonds, non-covalent interactions are reversible and highly responsive to external physical or chemical stimuli (e.g., heat and solvent). H-bonds, π−π stacking, van der Waals forces and other non-covalent interactions have lower energy, and is easy enough to induce assembly and disassembly when using to an external stimulus. A supramolecular fibrous LC network featuring heat recovery was employed to construct soft materials like physical gels [30,31].

Many supramolecular LC systems were formed between LC molecules, units or other components, and appeared in different new phases [32,33,34,35]. Different from conventional LC, these highly ordered supramolecular architectures would be paid much attention due the response ability, not the nature of LC (e.g., phase transition, thermal stability and spatial order). Therefore, supramolecular LC formed by non-covalent interactions provided a new route to design multifunctional and practical materials [36,37,38,39,40]. 

In supramolecular LC systems, H-bonds are a general option to position different components in a certain arrangement with enhanced intermolecular binding strength [32,41]. For aromatic LCs, π−π conjugation can also cause these molecules to form well-organized supramolecular assemblies [42,43,44]. Other non-covalent interactions, like halogen bonds, ionic bonds and π−π conjugation, have their own unique way of constructing multifunctional LC supramolecules [45,46,47,48,49]. When combined with electron-donating groups or systems, LCs containing an electrophilic halogen atom can act as a acceptor to form halogen-bonded supramolecular LCs [48,50,51,52]. As for ionic LCs, they were formed by the self-assembly of ions in solution, and behaved with different self-assemblies and LC phases upon different solvent inductions [49,53].

Besides, non-covalent interactions can even boost inorganic materials with LC molecules forming nanoparticle–LC systems. Not surprisingly, graphene, carbon nanotubes and other multifunctional carbon nanomaterials containing large π-conjugated structures are easily assembled with LC molecules. For example, the nematic matrix, like 5CB and 8CB, can align carbon nanotubes to form long-range orientational ordered structures with a grooved surface or magnetic or electric field [54,55]. Additionally, carbon nanotubes can modify the LC’s anisotropy in optic and dielectric anisotropy [56,57,58]. Monolayer graphene flakes can provide the parameter of orientational order to enhance the dielectric anisotropy in the nematic LC of MLC-15600-100 [59]. It has also been reported that the presence of graphene flakes can accelerate the electro-optic response in a nematic LC [60]. H-bonds were also used as a linker to combine LC molecules and nanoparticles into a supramolecular whole [61]. For example, highly specific H-bonding interactions improved the dispersibility and compatibility of the ZrO_2_ nanoparticles in supramolecular nematic LC nanocomposites [62].

## 4. Hydrogen-Bonded Supramolecular Liquid Crystal

H-bonds are an ideal non-covalent interaction for the construction of supramolecular architectures [39,63,64,65,66,67]. Once a donor (D) with an available acidic hydrogen atom interacts with an acceptor (A) carrying available non-bonding electron lone pairs, H-bonds arise and, therefore, are endowed with high selectivity and directionality [39]. Importantly, H-bonds are easily affected by solvents, salts, ions, temperature and so on, so they are viewed as key to the controllability of H-bonded supramolecular systems. As a tool to assemble supramolecular architectures, H-bonding can bring positive effects to LC, e.g., to generate new phases, expand the phase temperature range, improve thermal stability and so on.

**Figure 1:**
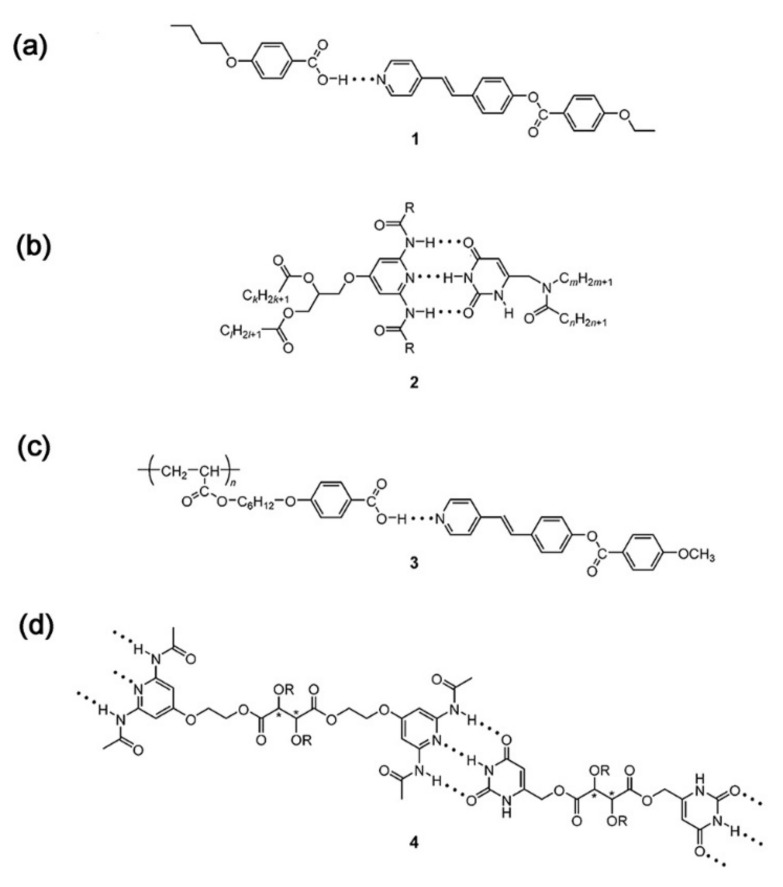
Supramolecular hydrogen-bonded liquid crystal (LC) molecules: (**a**) Low molecular weight complex by Kato and Fre’chet in 1989. Reprinted with permission from reference [80]. Copyright (1989) American Chemical Society. (**b**) Low molecular weight complex by Lehn and coworkers in 1989. Reprinted with permission from reference [81]. Copyright (1989) Royal Society of Chemistry. (**c**) Side-chain polymer by Kato and Fre’chet in 1989. Reprinted with permission from reference [82]. Copyright (1989) American Chemical Society. (**d**) Main-chain polymeric complex by Lehn and coworkers in 1990. Reprinted with permission from reference [83]. Copyright (1990) John Wiley and Sons.

**Figure 2:**
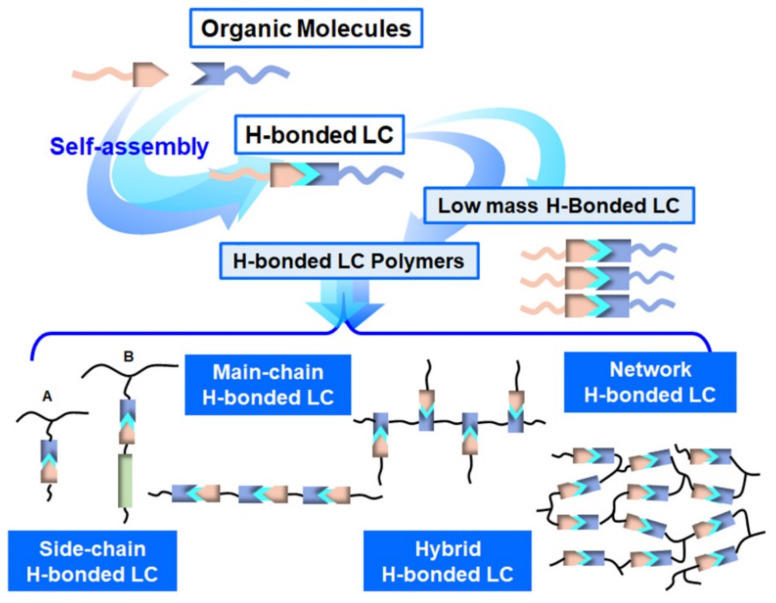
Classification of H-bonded supramolecular LCs.

As for H-bonded supramolecular LC (see Figure 1), the common structures are formed through benzoic acid, carboxylic acids, pyridyl and others [68,69]. In the first example of H-bonded supramolecular LC complexes, the LC state was formed by intermolecular H-bonds within benzoic acid derivatives [70]. According to the number of hydrogen bonds, H-bonded supramolecular LCs are classified into two categories, single H-bonded [71,72] (see Figure 1a) and multiple H-bonded ones [73,74,75] (see Figure 1b). Furthermore, especially for LC polymers, side-chain (see Figure 1c) and main-chain H-bonded supramolecular LCs (see Figure 1d) are the most common. In addition, there are some hybrid and network H-bonded supramolecular liquid crystals [76,77,78,79]. Figure 2 shows the formation and brief classification of H-bonded supramolecular LCs.

Complex 1 (see Figure 1a) consists of 4-butoxybenzoic acid (4BA) and trans-4-[(4-ethoxybenzoyl)oxy]-4’-stilbazole (2Sz) with one H-bond, featuring sematic and nematic phases. The mesomorphic range of Complex 1 was extended to 102 °C, and a new smectic phase appeared between 136 °C and 160 °C, but each component showed only a nematic phase [80]. Complex 2 (see Figure 1b) was formed with three parallel H-bonds between the uracil and 2,6-diaminopyridine groups, with a columnar hexagonal LC mesophase [81]. There was no mesomorphic phase of these pure compounds. Fortunately, the mesophase became observable only if long enough aliphatic chains were introduced into the uracil derivatives. Side-chain polyacrylate was used as framework to build up a special supramolecular LC through hydrogen bonds between its pendant pentoxybenzoic acid groups and guest stilbazole ester (see Figure 1c) [82]. The mesomorphic range of the mixture with the determined ratio (polyacrylate: stilbazole ester = 1:1) reached 112 °C, while the corresponding ranges of each component were 15 and 48 °C, respectively. It is likely that this strong enhancement on the mesophase is due to the formation of hydrogen-bonded complexes. On the other hand, supramolecular main-chain “polymers” were obtained with triply hydrogen-bonded complementary pairs between uracil and 2,6-diacylamino-pyridine (see Figure 1d) [83]. Under different conditions, these 1:1 mixtures showed special optical textures (e.g., stretched and helically wound fibers). 

One of the obvious traits of these supramolecular H-bonded mesogens is high thermal stability. The stabilization effect greatly contributes to the ordered condensed state of hydrogen-bonded supramolecular LCs. Another merit is the unique reversibility during dynamic formation and breaking, compared with covalent-bonded LC molecules. Both of them provide a specific idea for designing new LC structures [16,33]. Naturally, H-bonded supramolecular LCs have been rapidly developed into a flexible, facile and cost-effective approach for novel smart materials [84,85,86].

In this review article, we describe topics on the recent progress of H-bonded supramolecular liquid crystal, which consists of assemblies among organic LC molecules, or between inorganic materials and organic LC molecules. We will discuss molecular structures and the relationships between molecular self-assembled structures and functions, especially the formation, mechanism and advantage of hydrogen bonds. In addition, we focus on some H-bonded LC systems which showed a new LC phase, wider phase transition temperature, better mechanical properties, “reversible” deformation behaviors, wider reflection area or other better performance properties with some external stimuli, for example, light, pH, thermality and humidity.

## 5. Hydrogen-Bonded Supramolecular Self-Assemblies of Organic Liquid Crystal Molecules

As mentioned above, H-bond sensitivity to an external stimulus gives LC complexes diverse response functions (e.g., light, pH, humidity and heat). Here, H-bonded supramolecular LCs are classified into a few representative types according to their stimulus-responsive capability.

### 5.1. Photo-Responsive Type

The key is to introduce light-sensitive chromophores into H-bonded supramolecular LC systems [87]. Molecules with photo-isomerized azobenzene groups are a fine example that can trigger self-assembly and disassembly by intermolecular H-bonds [88,89,90,91]. In these cases, the azobenzene groups underwent reversible trans–cis transformation under ultraviolet (UV) and visible (Vis) light irradiation. The azobenzene isomers proceed with a geometrical change on the molecular level [92,93,94,95,96] 

In particular, photo-driven actuators have been rapidly upgraded to H-bonded supramolecular LC polymers (LCPs), including azobenzene-containing side-chain [91] and main-chain LCPs [89]. Their outstanding self-healing and photoinduced deformable properties keep attracting more and more attention [97]. As for H-bonded LCs based on azobenzene molecules or a polymer, proportion of H-bonds and the resulting LC phase in the LC complex can be tuned by photoisomerization [98]. 

**Figure 3:**
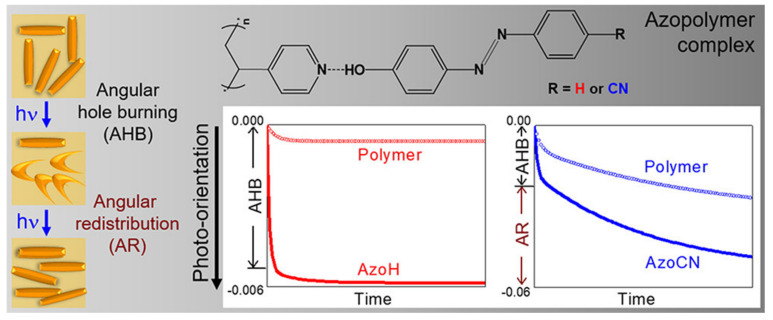
Supramolecular complexes of poly(4vinylpyridine) (P4VP) with 4-phenylazophenol (A_H_, R = H) and 4-hydroxy-4′-cyanoazobenzene (A_CN_, R = CN), and photoinduced orientation as a function of time during orientation (laser on) of P4VP/A_CN_ (100%) and P4VP/A_H_ (100%). Reprinted with permission from reference [103]. Copyright (2018) American Chemical Society.

Supramolecular side-chain LCPs are usually formed by H-bonds between hydroxyl and cyano groups [90,99,100,101,102]. Wang et al. employed 4-hydroxy-4′-cyanoazobenzene and poly(4vinylpyridine) with 4-phenylazophenol (P4VP) to construct H-bonded supramolecular azobenzene complexes (see Figure 3) [103]. Along with an increasing molar ratio of azobenzene groups to the pyridine ring of P4VP repeat units (F_A_), H-bonding complexation, as well as the band intensity in infrared spectra, were enhanced. For P4VP/AzoH, angular hole burning (AHB) did not allow a stable orientation, or orientation transfer from azobenzene to pyridine through H-bonds, which led to a decrease in the photo-orientation efficiency at a high azobenzene content. However, for P4VP/AzoCN, the angular redistribution (AR) made a positive contribution to the photo-orientation at a high azobenzene content. This may be caused by the emergence of an LC mesophase, as well as the much higher dipole moment of AzoCN compared to AzoH. Obviously, H-bonds regulated the optical photo-orientation of this supramolecular polymer. Koskela et al. applied H-bonds between guest azobenzene units to stabilize the photoinduced birefringence of a bisazo-polymer. These resulting H-bonded complexes play a leading role in this stabilizing effect, owing to their large length-to-diameter ratio and low side-chain mobility compared with the corresponding monoazo-functionalized polymers [91]. Yu et al. fabricated supramolecular LC polymer microparticles through H-bonds between an azopyridyl polymer and a series of dicarboxylic acid compounds [104]. Photoinduced deformation occurred with LC phases for the assembled microparticles, whereas no changes in morphologies were observed. Importantly, in the LC composite, H-bonds make those microparticles more stable upon photo irradiation.

**Figure 4:**
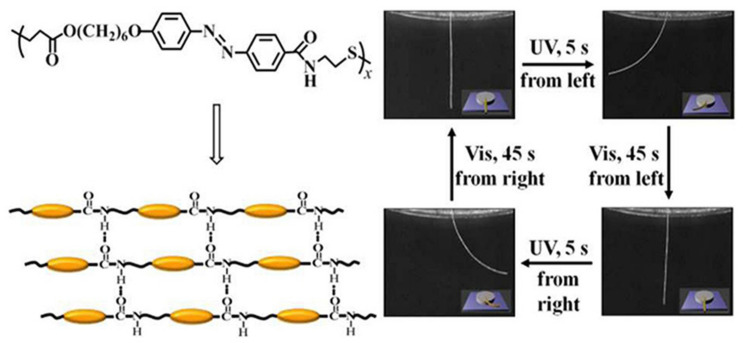
H-bonding self-assembly and photoinduced deformation upon irradiation with 365 nm UV light (150 mW cm^−2^) and visible light (λ > 510 nm, 120 mW cm^−2^) at 25 °C. Reprinted with permission from reference [107]. Copyright (2017) Royal Society of Chemistry.

Compared with side-chain ones, main-chain LCPs favor larger deformation upon some irradiation levels owing to their better mechanical properties and stronger chain anisotropy [105]. Typically, in physically crosslinked supramolecular fibers, the secondary amino or amide groups formed H-bonds, and other azo groups completed photoinduced deformation upon UV irradiation (wavelength: 365 nm) at 40 °C [106]. Subsequently, among azo polymer chains, strong H-bond interactions between amide groups can bring higher thermal stability, a lower glass transition temperature and a wider crystalline phase temperature range. Additionally, H-bonds play a vital role in photomobility for the much higher mechanical strength of azo polymers [107]. Figure 4 shows the inherent molecular self-assembly through H-bonds, and the resulting macroscopic deformation of these Azo-PEA-6 fibers upon exposure to UV and visible light, respectively. The response time for the LC fiber bending decreases rapidly with the increase in both the UV light intensity and the ambient temperature, and finally becomes almost unchanged with a further increase. Clearly, higher UV light intensity or temperature can accelerate reversible isomerization of the backbone azobenzenes. However, another polymer similar to Azo-PEA-6, in which the ester bond replaced the original amide bond, showed no photodeformation upon exposure to UV or visible light. This verified the decisive role of the H-bond-crosslinked networks in the recycled photomobility. Better thermal stability, a lower glass transition temperature and a wider range of the smectic phase appeared and developed from H-bonding in this structure.

Moreover, chiral nematic mesoporous organosilica (CNMO) films were constructed via H-bonding self-assembly between hydroxyl and cyano groups, in which a columnar nematic structure with broad range of phase transition was formed inside the pores. Since H-bonding can adjust the orientational order of the mesogens, the photonic properties of the chiral nematic mesoporous host can be tuned by heating or UV irradiation [108]. As soon as it is irradiated by UV light at 365 nm for 5 s, a weakened birefringence of the LC guest was observed, showing photochromic behavior of hybrid materials. Interestingly, H-bonded supramolecular LC composites were applied to build up fluorescence-responsive materials [109]. Compared to the LC composites without H-bonds, the H-bonded LC composites behaved with a significant shift of the emission band to the long-wave region in their fluorescence spectra upon irradiation. After annealing treatments, the irradiated H-bonded LC composites underwent a decrease in dichroism values, but the one without H-bonds experienced a significant increase. Given the heat sensitivity of H-bonds, this may be attributed to the destroyed orientation of H-bonded supramolecular LC composites during annealing. Similarly, this kind of LC composite can also reorganize to perform photoinduced orientation once exposed to UV light [110]. The internal LC alignment direction can be regulated by exposure energy.

### 5.2. pH-Responsive Type

Harris et al. reported a H-bonded monodomain LC nematic network that can conduct a dramatic reversible bending deformation in response to small changes in pH value [111]. Here, H-bonds appeared within the carboxylic acid groups of acrylate monomers. When exposed to KOH solution, this LC film underwent a reduction in H-bonding to disrupt the nematic cores of the mesogenic network, followed by anisotropic deformation. Below the pH threshold, the LC network expansion was modest, along with little detectable anisotropy. Above the pH threshold, the expansion rate in the perpendicular direction significantly exceeded that in the parallel direction, along with H-bond breakage and loss of ordering.

**Figure 5:**
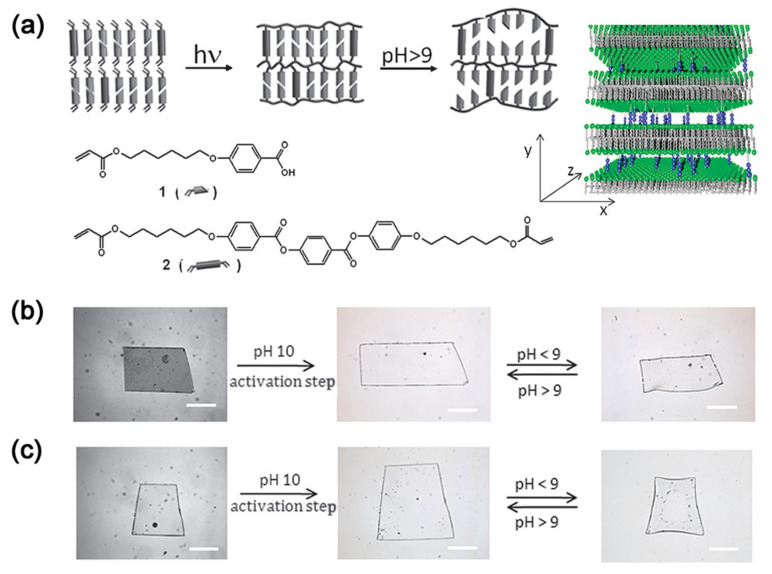
(**a**) Schematic representation of the formation of a smectic hydrogel based on LC monomers, and the swelling at high pH value. The 3D figure illustrates the nanopores formed by hydrogen bridges. (**b**) Swelling and deswelling of thin films of a planar-aligned smectic network (**c**) and a homeotropic smectic network (scale bar corresponds to 500 μ). Reprinted with permission from reference [113]. Copyright (2012) Royal Society of Chemistry.

Afterwards, Dirk J. Broer’s group adapted H-bond-bridged smectic networks to fabricate well-ordered nanoporous membranes [112,113]. This kind of smectic well-ordered polymer nano-membrane can be switched between an open and a closed state by modulation of the pH (see Figure 5a). At high pH values, the smectic membrane is in the open state owing to the destruction of interlamellar H-bond bridges. Once those bridges were broken, the smectic networks generated periodic lateral pores in both a planar alignment and homeotropic alignment, to absorb water rapidly by spontaneous permeation. Of course, a reversible swelling behavior also occurs as the ambient pH value changes (see Figure 5b,c) [113].

Besides, the control of H-bonds is also viewed as a common method to improve the sensitivity of the helical supramolecular structure. InChen et al.’s paper, the selective reflection band (SRB) of the cholesteric LCP film above pH 7 showed an obvious red shift with increasing pH values, but hardly changed at pH 7 or below pH 7. This pH sensitivity may be attributed to the breakage of H-bonds formed by isonicotinate and acrylates above pH 7 [114].

### 5.3. Thermo-Responsive Type

Cholesteric LCs (CLCs) possess outstanding reflection characteristics and are widely used in colorimetric sensors and display devices [115,116,117,118]. This characteristic comes from changes of helical pitch (*p*), namely, uniformly periodical layer deformations. In general, *p* is defined as the distance in which CLC molecules rotate 360° around the helical axis to propagate light [119]. The corresponding wavelength of the maximum reflection (λ_0_) is calculated by the following equation:

$$\lambda_{0}=p\;\times \;\overset{-}{n}$$

where $\overset{-}{n}=(n_{e}\;+\;n_{0})/2$ , namely, the average refractive index. So, the photonic band gap (PBG) width of a conventional CLC is equal to the product of *p* and Δ*n*. 

In other words, changes of PBGs in periodical helical orientation will bring about changes of CLC reflective colors. It was demonstrated that controlling the breaking and forming of H-bonds in CLCs can cause PBG changes [120]. In this study, the CLC composites comprised a nematic LC molecule, a H-bond chiral dopant (HCD) and a series of cholesteryl esters. When heated above 60 °C, the H-bonds in the HCDs were broken and therefore two new chiral dopants comprising an initial proton acceptor and proton donor were obtained with a resulting blue shift of the reflective color.

On the other hand, H-bonded chiral monomers (HBCMs), including cholesteryl isonicotinate as a proton acceptor and 4-(6-acryloyloxyhexyloxy) benzoic acid as a proton donor, were introduced to build up the CLC system [121]. While the temperature rose from 25 to 75 °C or when applying a voltage, many H-bonds of the HBCM were broken, and then the helical twisting power value (HTP) of both the HBCM and cholesteryl additives increased to generate a blue shift of the reflection band from 780 to 540 nm. This variation made this system’s color change from the initial red to green. Similarly, a CLC composite self-assembled by H-bonds of chiral molecules exhibited an unusual red shift of selective reflection band due to heating [122]. Here, he temperature increase makes the internal H-bonds break, which decreases the HTP value, viz., increases *p*. Otherwise, the opposite transition will happen.

On top of these interesting examples, Schenning et al. proposed an optical time–temperature steam sensor based on crosslinked supramolecular CLC films [31]. The side carboxyl groups of the acrylic monomers interacted to form H-bonds inside. While the CLC film was heated up above the isotropic temperature (*T_iso_*), the order loss of the internal photonic structure accompanied those broken H-bonds to bring about a transparent coating in the isotropic phase. After cooling down below *T_iso_* for 20 min, a white scattering film was obtained due to H-bond recovery. 

**Figure 6:**
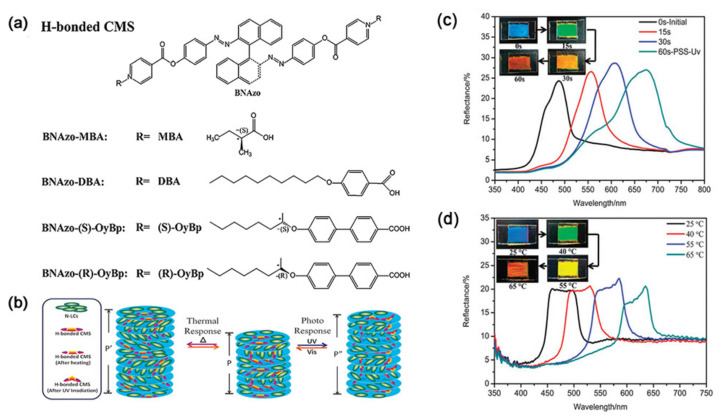
(**a**) Chemical structures of H-bonded chiral molecular switch (CMS) and proton donors. (**b**) Schematic diagram of the helical superstructure and the corresponding switching mechanism of H-bonded CMS in a nematic liquid crystal (NLC) host reversibly tuned by UV/Vis light and heat. The photographs and reflection spectra of 8.1 wt% BNAzo-MBA in SLC1717 in 10 mm thick planar cells under UV irradiation (**c**) and at various temperatures (**d**). Reprinted with permission from reference [123]. Copyright (2015) Royal Society of Chemistry.

In general, a thermo-responsive hydrogen-bonded CLC composite can also respond to other stimuli such as light and electricity. Jin et al. introduced H-bonded chiral molecular switches (CMSs) and photosensitive domains (e.g., azobenzene) into CLC films to construct a dual photo- and thermo-responsive supramolecular system [123]. The HTP was switched by temperature and UV/Vis light for different reflective colors (see Figure 6). Here, the thermo-modulation of the H-bond interaction between donors and acceptors plays an important role for the CLC composite. In the four H-bonded CLC mixtures containing different chiral molecular switches, all the reflection bands exhibited a red shift under UV irradiation or heat treatment. 

### 5.4. Humidity-Responsive Type

Humidity-responsive LC materials have also been investigated a lot for actuators and sensors. Here, the sensitivity of H-bonds to moisture pushes H-bond supramolecular LC systems to play a vital part.

**Figure 7:**
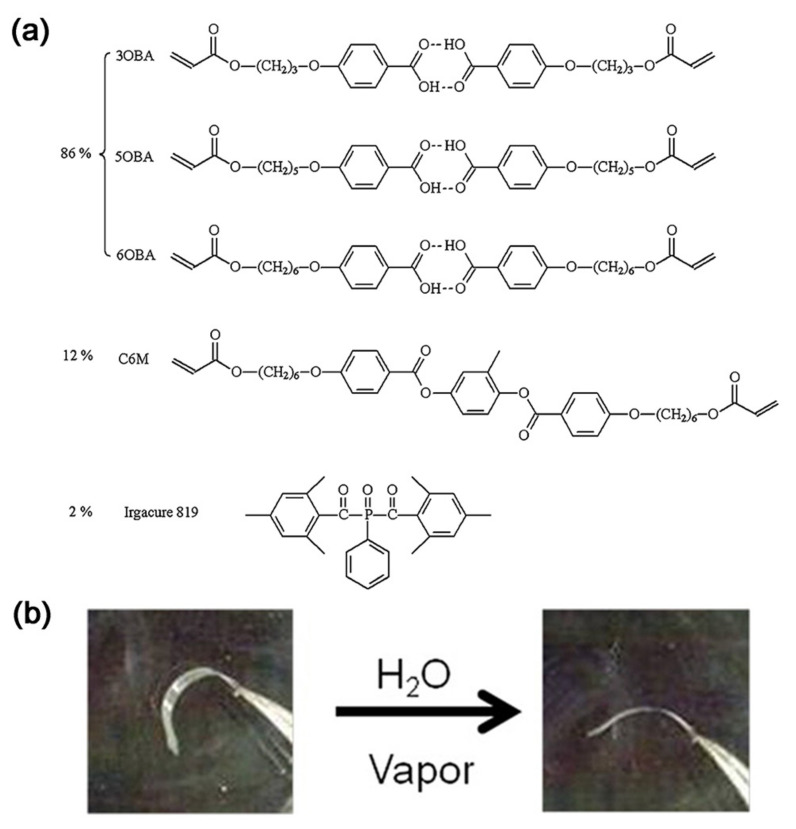
Formula of the LC mixture (**a**) and the humidity-responsive deformation (**b**). Reprinted with permission from reference [125]. Copyright (2013) American Chemical Society.

Harris et al. developed a supramolecular H-bonded LCP network and converted it into a polymer electrolyte under alkaline conditions. At this point, the inner H-bonds disappeared, and this salt network became much more hygroscopic than the crude one. Once in contact with moisture, it swelled to produce macroscopic deformation [124]. Then, Schenning et al. fabricated a humidity-responsive supramolecular bilayer system via H-bonding between the side carboxy groups of the acrylate monomers (see Figure 7). This film was first treated with an alkaline solution for H-bond breakage. After drying, it showed a reversible deformation with spontaneous response to ambient humidity [125]. Similarly, another LC hydrogen-bonded actuator was made by the same alkaline treatment. The actuator was straight at a humidity level of 75%, but strongly bent at a humidity level of 15%. Furthermore, when a base treatment was selectively performed on the specific surface regions, like patterning, the film underwent a violent deformation, with the bending angle varying from −45° to +45° [126].

Additionally, a H-bonded cholesteric LCP was prepared by treatment with 0.05 M KOH solution for a printable and optical humidity-responsive sensor. Here, most of the H-bonds formed by acrylate monomers were broken, and then the LCP was saturated with water to yield a red-reflecting film. A fast reversible change of the reflection color between green and yellow happened when it was exposed to water [127].

### 5.5. Other Stimulus-Responsive Types

In recent years, the detection of organic vapors has been viewed as a hotspot in the field of sensors. Particularly, LC materials have also been adopted for organic vapor sensors [128,129,130,131], even supramolecular H-bonded LC materials [132]. It has been reported that a H-bond-bridged cholesteric LCP network was constructed to distinguish between ethanol and methanol as an optical sensor material [133]. In this photopolymerizable LCP film, H-bridges were formed between the side carboxylic groups of acrylic acids monomers. Once these H-bonds were broken and activated using an alkaline solution, the network became more porous on a molecular length scale to stimulate analyte absorption. Typically, those hydroxyl groups of alcohol molecules may interact with the activated carboxylic moieties to produce a higher extent of expansion than crude H-bridged networks, followed by a different reflective color. Ethanol molecules showed a larger red shift compared to methanol molecules.

**Figure 8:**
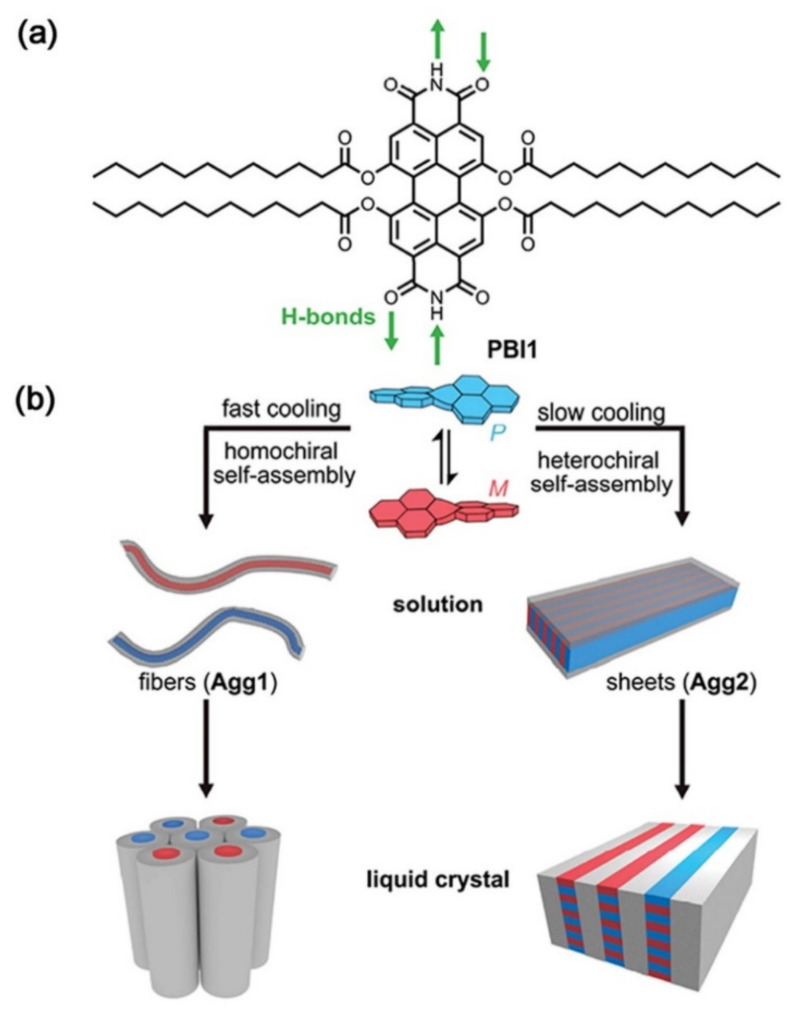
(**a**) Chemical structure of PBI1. (**b**) Schematic illustration of homochiral fibers (Agg1) and heterochiral sheets (Agg2) self-assembled by cooling PBI1 solution (90 °C) with 10 and 0.6 K/minute, respectively, and their subsequent organization into columnar or lamellar LCs. Reprinted with permission from reference [134]. Copyright (2020) John Wiley and Sons.

Hecht et al. adopted perylene bisimide (PBI1) to build up columnar and lamellar LC systems into fibers and sheets by homo- and heterochiral H-bonded self-assembly upon different cooling rates, respectively (see Figure 8) [134]. While the helical structure of Agg1 is formed by a homochiral arrangement of the respective P- and M-atropo-enantiomers, the sheets of Agg2 are formed by an alternating heterochiral arrangement of the two. In a hot solution, PBI1 self-assembles into two different aggregates, with purple fluorescence at a fast cooling rate and red fluorescence at a slow cooling rate. The intermolecular hydrogen bonds provide the possibility for the formation of these two different assembled structures of fibers and sheets.

**Figure 9:**
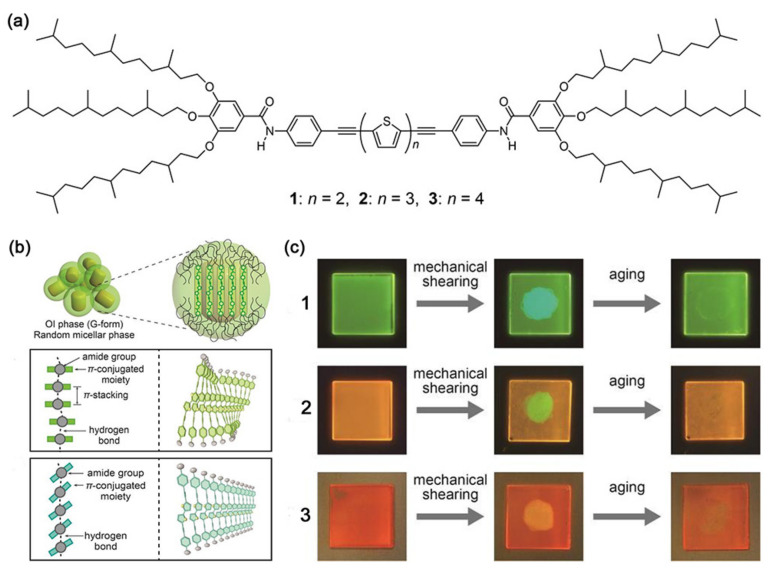
(**a**) Chemical structures of LC compounds 1–3. (**b**) Schematic illustrations of compound 1 assembly, the optically isotropic (OI) phase (G form) and the unidentified mesophase (M) phase (BG form). (**c**) Luminescence images of compounds 1–3 under UV irradiation (365 nm) before (left) and after shearing (center), and after aging at ambient temperature (right). Reprinted with permission from reference [135]. Copyright (2016) Royal Society of Chemistry.

Kato et al. fabricated a shear-responsive supramolecular H-bonded LC film containing bi-, ter- and quarter-thiophene moieties [135] (see Figure 9a). All these compounds exhibited various reversible luminescent colors under the action of shearing force. As shown in Figure 9c, compounds 1–3 exhibited reversible luminescent color changes at ambient temperature, for compound 1 from green to blue–green, compound 2 from orange–yellow to yellow–green, and compound 3 from red–orange to yellow. These unique luminescence phenomena were attributed to reducing of the luminescent cores, in which the H-bonds between π-conjugated moieties transformed into linear modes to trigger the phase transition, e.g., the OI–M phase transition in compound 1 (see Figure 9b). Once shear force was applied, these phase changes appeared. Similarly, another shear-responsive LC film using benzodithiophene molecules was reported [136]. At an applied shear press of 2.4 × 10^5^ Pa (shear strain: 390%), this film showed a luminescent color change from yellow to sky blue, due to a phase transition from a rectangular columnar to a metastable optically anisotropic mesophase. Interestingly, once shear stopped, the film emitted a blue–green luminescent along with reaggregation of LC molecules. Besides, it showed a reversible color transition at 150 °C. In the two examples mentioned above, all these structures and colors were greatly dependent on the balance between intermolecular H-bonds and π–π interactions.

**Figure 10:**
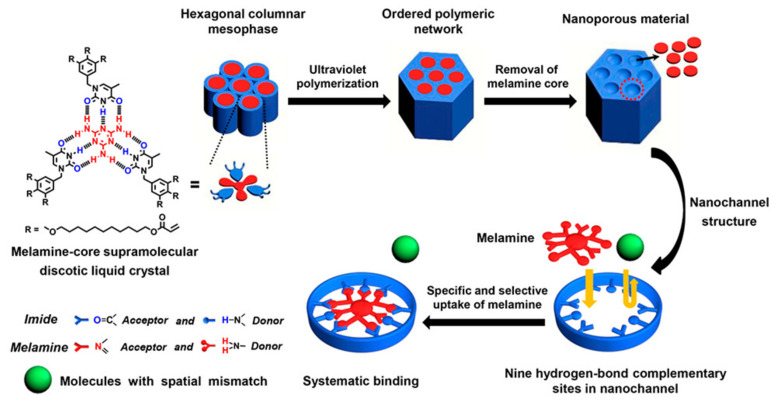
Schematic illustration of the design and fabrication of supramolecular discotic LC polymeric material for specific and selective uptake of melamine. Reprinted with permission from reference [142]. Copyright (2020) American Chemical Society.

Besides, supramolecular H-bonded LC complexes have been applied a lot in nanoporous materials for selective molecule or ion adsorption [137,138,139]. For example, melamine has not only attracted extensive attention since the milk powder incidents, but has also become more and more important in the food industry. Yang et al. prepared supramolecular H-bonded discotic LCs with a melamine core [140]. Here, five H-bonded complementary functional groups, i.e., benzoic acid, cyanuric acid, homophthalimide, succinimide and thymine derivatives, were involved. By comparison, the imidodicarbonyl unit was the best. Finally, different columnar mesomorphic orders (e.g., hexagonal, rectangular and square columnar phases) were obtained by adding functional thymine or succinimide units, and changing the molar ratios of melamine to those of H-bonded complementary compounds. Subsequently, this group reported a nanoporous supramolecular H-bonded LC polymer based on a melamine/thymine derivative for the specific recognition and absorption of melamine (see Figure 10) [141]. It showed a stable and recyclable absorbability of melamine within a wide pH range (pH 4~10). On the other hand, these nanoporous supramolecular H-bonded materials were constructed using melamine as a template, but also kept the columnar hexagonal order after the removal of the template. Different pore sizes brought about different absorbabilities. In another similar work, new discotic LCs, i.e., columnar hexagonal H-bonded complexes, were fabricated by the self-assembly of a melamine core and a tris(triazolyl)triazine derivative [142]. As a template, this LC complex was treated with polymerization and the removal of the melamine core, to obtain two nanoporous polymers with different pore diameters. After base treatment, both of the systems could selectively adsorb cationic dyes by electrostatic interaction.

## 6. Hydrogen-Bonded Supramolecular Self-Assemblies of Inorganic Materials and Organic Liquid Crystal Molecules

Besides organic molecules, inorganic materials with high anisotropy have also become a good choice of dopant to improve the electro-optical performance of LC systems. In recent years, the LC-mediated assembly of nanoparticles (NPs) has been developed a lot into new tunable meta-materials for electronic, photonic and optical applications [143,144,145]. In many systems, H-bonds were a key linker to combine LC molecules and NPs together, leading to better stimulus-responsive performance [146]. 

In general, the compatibility of inorganic NPs with LC is a huge challenge in this field. Surface modification is the most common solution for good compatibility and dispersibility [62,147,148]. Most oxide NPs generally aggregate into primary particles by H-bonds between the hydroxyl groups on the NP surface [149]. Similarly, carboxyl acid group-containing LC molecules can induce the aggregation of pendant carboxylic acid group-decorated NPs via intra- and inter-particle H-bonds. In order to avoid NP aggregation, the NP surface can be modified to construct H-bonded supramolecular LC without end carboxyl groups, and to promote NP–LC coupling [62].

**Figure 11:**
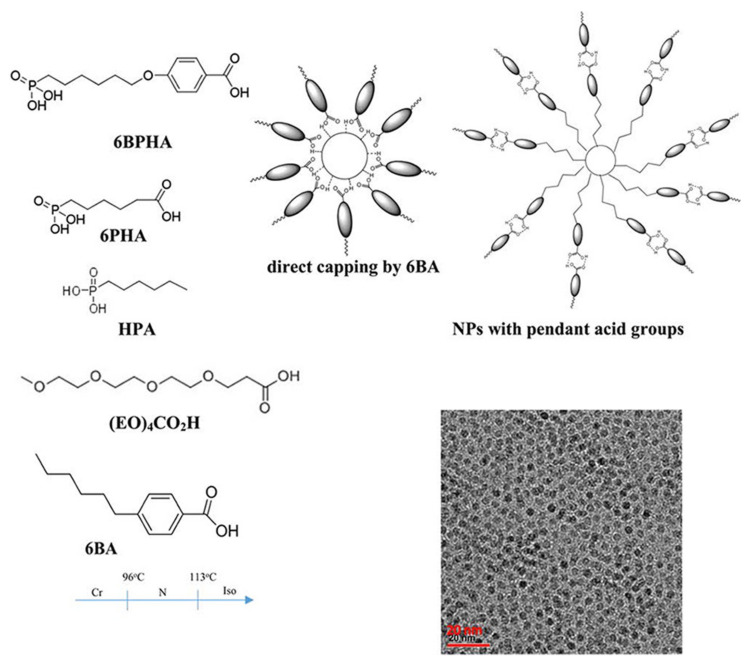
Schematic diagrams of LC molecules, ligands, functionalization types and proposed H-bonding interactions with the LC matrix along with a TEM image of the ZrO_2_ nanoparticles. Reprinted with permission from reference [61]. Copyright (2016) American Chemical Society.

Therefore, H-bonds can also be introduced to form a stable dispersion of NP–LC composites. Figure 11 shows a H-bonded LC nanocomposite comprising 4-(n-hexyl)benzoic acid (6BA) and zirconia (ZrO_2_) NPs capped with the diacids 6-phosphonohexanoic acid (6PHA) and 4((6-phosphonohexyl)oxy)benzoic acid (6BPHA), respectively [61]. Obviously, there were two different pendant acid groups, i.e., carboxylic and oxybenzoic acid groups, on the surface of ZrO_2_ NPs. Particularly, the ratio control of the two surface groups can reduce undesired interactions within NPs, which is due to the high selectivity of H-bonds. Roohnikan et al. prepared a few model LC mixtures comprising trans-4-n-butylcyclohexanecarboxylic acid (4-BCHA), 4-hexylbenzoic acid (6BA) and 4,4ʹ-bipyridine, and analyzed their molecular-level H-bond interactions with ZrO_2_ NPs systematically by solid-state ^1^H and ^13^C nuclear magnetic resonance (NMR) and Fourier transform infrared spectroscopies (FT-IR) [150]. The solid-state 1H chemical shifts for the model complexes indicated that the carboxyl–pyridine linkages of 6BA to the ZrO_2_-pyridine NPs were stronger than the ones of 4-BCHA. When 6BA was hybridized with BPy, the acid proton of 6BA underwent a large shift from 14 to 16 ppm. In addition, the heterodimer formation of pyridine groups can also be demonstrated by peak change.

Furthermore, new phenomena were found inside H-bonded supramolecular LC/ZnO nanospike composites [151]. By polarized optical microscopy, different phases were observed, but also lower temperature transitions, fast response times and high switching rates compared to the pure LC samples. This was ascribed to the highly ordered assembly, especially internal π–electron stacking and dipole–dipole interactions between LC molecules and ZnO nanospikes. 

Katranchev et al. introduced carboxyl-functionalized single-walled carbon nanotubes (SWCNTs) into alkyloxybenzoic acids (8OBA), to form a stable H-bonded LC composite [152]. Ezhov et al. utilized CdS nanorods (NRs) and poly(4-(nacryloyloxyalkoxy) benzoic acids to produce a smectic C H-bonded nanocomposite. The optical properties of this system were demonstrated to be dependent on the extent of CdS NR microphase separation [153]. Similarly, Shandryuk et al. prepared another H-bonded LC nanocomposite using CdSe quantum dots (QDs) and poly[4 -(n-acryloyloxyalkoxy)] benzoic acids. The nanolayers of the H-bonded side-chain LC polymers on the surface of CdSe QDs make the resulting QD–LC system more stable and induce the QDs to become arranged in the smectic layer [146]. Due to confinement to mesoporous aluminosilicate molecular sieves (AlMCM-41), H-bonded supramolecular LC composites showed special phase behavior and structural organizations [154]. Most of molecular H-bonds were broken, and then the loaded monomeric 4-HBA and 4-BCA were chemisorbed to the AlMCM-41 pore surface by bidentate coordination bonds to aluminum cations. Naturally, the crude LC phase disappeared, showing a wide temperature range of 10–120 °C.

## 7. Summary

This review mainly summarized the recent advances in H-bonded supramolecular LC systems. Here, the formation mechanism and the inherent nature of supramolecular LCs were emphasized to highlight the enhancement of noncovalent interactions in molecular anisotropy and stimulus-responsive capability. Particularly, basic directionality, selectivity and reversibility make H-bonds the best option to build supramolecular LC architectures. Some external stimuli easily break H-bonds with the LC phase transition, and further induce property changes, and even macroscopic deformation. All of these merits make H-bond supramolecular LC systems more functionalized and intelligent. Light, heat, pH value, humidity and other physicochemical factors were employed as H-bond switches to the structures and properties of supramolecular LC self-assemblies. Moreover, inorganic nanomaterials were also introduced to improve electro-optical performance for optoelectronics. In the foreseeable future, H-bonded supramolecular LC materials will keep developing more new functions and applications.

## 8. Future Directions

Good sensitivity to external physicochemical stimulus or conditions makes H-bonded supramolecular LC systems adjustable in structure and property, showing excellent controllability, but joint H-bonds are unstable. This is a great challenge for applications in which both controllability and stability must be taken into consideration.

1. Compatibility, including material, surface, interface, and cross-scale compatibility, is also viewed as a big barrier for supramolecular LCs, especially supramolecular LCs self-assembled by organic LC molecules and inorganic materials. Either shape and size, or surface groups and chemical properties, make huge differences between organic and inorganic components. They are not conductive to the overall compatibility of supramolecular LCs. Some effective solutions, e.g., surface modification, need to be performed during the construction of supramolecular LCs.
2. The existing applications of H-bonded supramolecular LCs still focus on reflectors, sensors and actuators. More functions and applications need to be developed.

## Data Availability

No new data were created or analyzed in this study. Data sharing is not applicable to this article.
